# The Reduced Effectiveness of Protected Areas under Climate Change Threatens Atlantic Forest Tiger Moths

**DOI:** 10.1371/journal.pone.0107792

**Published:** 2014-09-17

**Authors:** Viviane G. Ferro, Priscila Lemes, Adriano S. Melo, Rafael Loyola

**Affiliations:** 1 Departamento de Ecologia, Universidade Federal de Goiás, Goiânia, Goiás, Brazil; 2 Programa de Pós-Graduação em Ecologia e Evolução, Universidade Federal de Goiás, Goiânia, Goiás, Brazil; University of Western Ontario, Canada

## Abstract

Climate change leads to species' range shifts, which may end up reducing the effectiveness of protected areas. These deleterious changes in biodiversity may become amplified if they include functionally important species, such as herbivores or pollinators. We evaluated how effective protected areas in the Brazilian Atlantic Forest are in maintaining the diversity of tiger moths (Arctiinae) under climate change. Specifically, we assessed whether protected areas will gain or lose species under climate change and mapped their locations in the Atlantic Forest, in order to assess potential spatial patterns of protected areas that will gain or lose species richness. Comparisons were completed using modeled species occurrence data based on the current and projected climate in 2080. We also built a null model for random allocation of protected areas to identify where reductions in species richness will be more severe than expected. We employed several modern techniques for modeling species' distributions and summarized results using ensembles of models. Our models indicate areas of high species richness in the central and southern regions of the Atlantic Forest both for now and the future. However, we estimate that in 2080 these regions should become climatically unsuitable, decreasing the species' distribution area. Around 4% of species were predicted to become extinct, some of them being endemic to the biome. Estimates of species turnover from current to future climate tended to be high, but these findings are dependent on modeling methods. Our most important results show that only a few protected areas in the southern region of the biome would gain species. Protected areas in semideciduous forests in the western region of the biome would lose more species than expected by the null model employed. Hence, current protected areas are worse off, than just randomly selected areas, at protecting species in the future.

## Introduction

The implementation and maintenance of protected areas is still the cornerstone of conservation actions [1]. However, due to broad-scale environmental changes which will potentially shift the distribution of suitable habitats for many species across the geographic space [2], [3], scientists have expressed concern that existing networks of protected areas might not be able to guarantee the long-term persistence of the species they are supposed to protect [4], [5].

Climate change poses a new challenge to the traditionally static way conservation planning is usually done, by forcing planning to become more dynamic [6]. Several species have already shifted their ranges to cooler regions, both in temperate regions and in the tropics, as a response to a warming climate [7]–[9]. Most solutions ofereded by conservation scientists and practitioners to deal with spcies' range shifts focus on the establishment of new protected areas that should cope with the effects of climate change on species distribution [6], [10]–[16]. However, the effectiveness of protected areas may decrease as they become climatically unsuitable for most species [5] and more suitable to invasive species [17], [18]. Climate-driven modifications in species composition within protected areas may disrupt species interactions [19] by altering ecosystem functioning [20]. Therefore, it is necessary to take into account forecasted changes in species distributions to evaluate the future effectiveness of protected areas [5], [6], [21].

The effects of climate change on species distributions have been generally inferred through ecological niche models (ENM) [22], also referred to as “bioclimatic envelope models” (BEM) [23] or “species distribution models” (SDM) [24]. Araújo and Peterson [25], Peterson and Soberón [26], and Rangel and Loyola [27] have all provided recent clarifications on their conceptual differences. However, different modeling methods and climate models may produce very different outputs, increasing uncertainties in projected distributions and their applicability to conservation efforts [3], [28]. In the last decade, new modeling techniques have been developed that take into consideration such impediments (e.g. model ensemble forecasting). Nonetheless, few studies have applied modern techniques to predict the distribution of invertebrates (but see Diniz-Filho et al., 2010a, b), despite an urgent need to evaluate the consequences of climate change to this hyperdiverse group in order to plan for its conservation [29].

Lepidoptera is the second richest order of insects, with 150,000 species recorded in the world [30]. Butterflies and moths are exclusive pollinators of many plant species [31], [32]. The vast majority of Lepidoptera larvae are herbivores, consuming almost all orders of gymnosperms and angiosperms as well as mosses and ferns [33]. Herbivory can influence the fitness [34], distribution, composition and abundance of plant species [35], as well as the rate of litter decomposition [36]. Lepidopterans are also important food items for arthropods (in particular spiders and other insects) and vertebrates (especially birds and bats). Changes in Lepidoptera diversity, abundance, phenology, distribution and assemblage composition driven by climate changes may affect ecosystem functions and services, species interactions, as well as the structure of plant communities and economic losses due insect infestation and pestilence [37].

Many studies have found that climate change will alter patterns of phenology, horizontal or vertical range (distribution and area), and the abundance of Lepidoptera species (reviewed in [38]). For example, Conrad et al. [39] studied the population dynamics of the tiger moth *Arctia caja* (Arctiinae) between 1968 and 1999 in Great Britain and observed a decrease of about 30% of abundance and proportion of occupied sites after 1984. Arctiinae (Erebidae) (classification following [40]), in particular, comprises almost 11,000 species of moths, of which about 6,000 are found in the Neotropics [41]. In Brazil, there are records for 1,391 species [42]. The Atlantic Forest has the richest Arctiinae fauna among all Brazilian biomes (1193 species) and approximately 40% of these species are endemic to this biome [43]. Arctiinae larvae feed on angiosperms and gymnosperms, as well as algae, lichens, and mosses [44]. Although Arctiinae are among the most polyphagous lepidopterans [44], the proportion of generalist species decreases toward the tropics. Many Arctiinae larvae and adults have conspicuous coloration, are diurnal and many adults form mimetic rings with Hemiptera, Hymenoptera, Coleoptera and unpalatable butterflies [45]. As well as exhibiting warning coloration, most of these moths are also toxic or unpalatable. Several secondary compounds were found in all stages of Arctiinae (eggs, larvae, pupae and adults), including pyrrolizidine alkaloids, which are generally sequestered from their host plants during the larval stage [45]. We choose this group because of its important roles in ecosystems and also because of their extremely high diversity in the region, so that we can run models using a great amount of data and keeping the taxonomic group narrow.

Here, we evaluated the current and future climatic suitability of protected areas located in the Atlantic Forest Biodiversity Hotspot (in Brazil) based on species' ecological niche models and diversity patterns of tiger moths (Lepidoptera: Erebidae: Arctiinae). More specifically, we addressed the following questions: (1) how will climate change affect the geographical pattern of Arctiinae species richness in the region? And (2) how does the spatial location of a given protected area determine if it will gain or lose species under different climate change scenarios?

We selected tiger moths as our case-study because they comprise a species-rich subfamily, are well represented in Brazilian collections (especially in the Atlantic Forest, which is the geographical area covered in this study) [42] and because there are several active researchers currently working with Neotropical Arctiinae. These researchers helped with the identification of some species and allowed for a nomenclatural update, incorporating recent occurrence records in our dataset.

## Methods

### Study region

**Figure 1:**
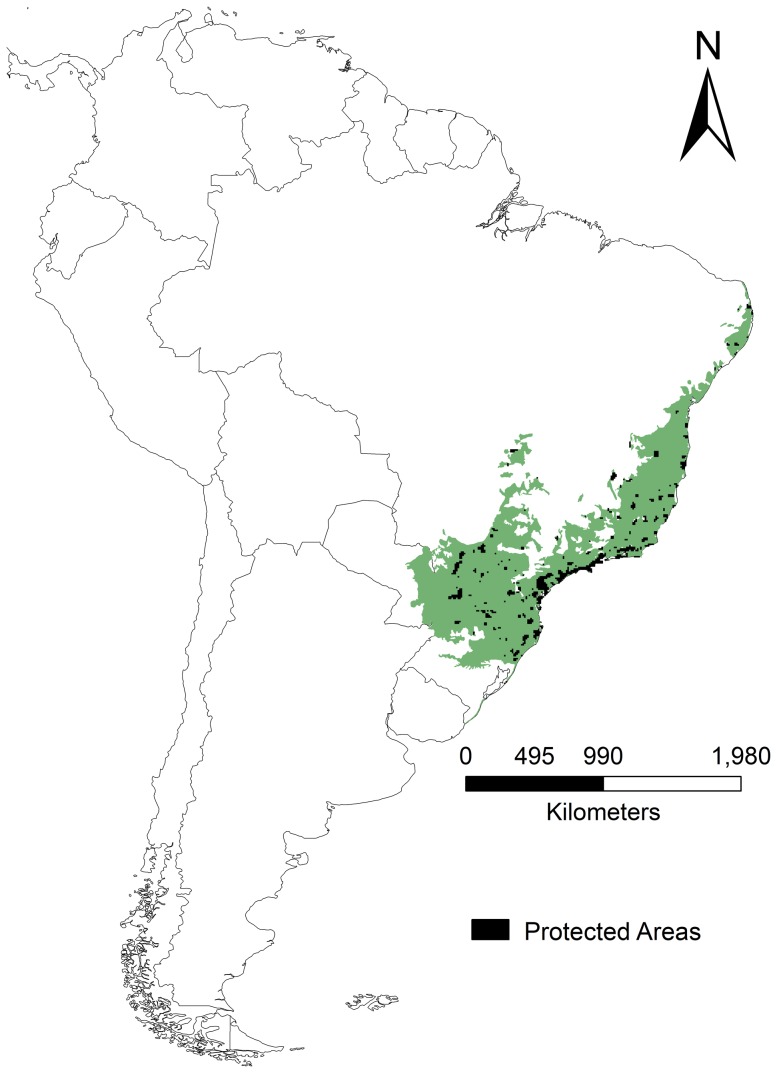
Original extent of the Atlantic Forest Biodiversity Hotspot in Brazil.

We focused our analyses on the Atlantic Forest Biodiversity Hotspot [46]. The Atlantic Forest originally covered around 150 million ha (Fig. 1) with heterogeneous environmental conditions. Its latitudinal range extends into tropical and subtropical regions, and its wide longitudinal range harbors differences in forest composition due to a diminishing gradient in rainfall from coast to interior [47]. Although the Atlantic Forest has high diversity and endemism (with more than 20,000 plant species, 261 mammal species, 688 bird species, 200 reptile species, 280 amphibian species, to name well-studied taxonomic groups), currently, only *ca.* 1% of the original forests are legally protected [47]. An effective reserve network, taking into account climate change to ensure the species persistence in the long-term, is therefore imperative to address conservation investment in appropriate sites.

### Ecological niche models

We obtained occurrence records for 703 tiger moth species inhabiting the Atlantic Forest from field surveys and museum records. Tiger moth records included the period of 1920 to2008. We overlaid these point-locality records for each species into an equal-area grid (10 km×10 km of spatial resolution) that covered the full extent of the Atlantic Forest. Then, we built a species by grid cell matrix, considering presences of species inside grid cells. Species with less than five occurrences were excluded to avoid model bias, and therefore, a total of 507 species were studied.

We obtained current climatic data from the WorldClim database (http://www.worldclim.org/current) and future climatic scenarios from CIAT (http://ccafs-climate.org). These future scenarios were developed by the Intergovernmental Panel on Climate Change (IPCC) Fourth Assessment Report (AR4). For each species, we modeled its distribution as a function of four climatic variables: annual mean temperature, temperature seasonality (standard deviation * 100), annual precipitation and precipitation seasonality (coefficient of variation). These variables represent interpolated climate data from 1950 to2000 [48]. For future climatic conditions we used the same climate variables for the year 2080, obtained from three Atmosphere-Ocean General Circulation Models (AOGCMs) of the A2 emission scenario (CCCMA-CGCM2, CSIRO-MK2.0 and UKMO-HadCM3), that were generated by application of delta downscaling method on the original data from IPCC's report. Data original resolution was 30 arc-seconds and both current and future climate variables were re-scaled to our grid resolution.

We used presence derived from species' occurrences and climatic variables to model species' ecological niche and project their distributions. As reliable absence data were not available, we fitted six presence-only modeling methods (which differ both conceptually and statistically [27]), grouped them into two separate sets (distance methods and machine-learning methods) and applied the ensemble forecasting approach within each set (see text below). Distance methods were Euclidian and Gower distances [49] and Ecological Niche Factor Analysis (ENFA) [50]. Machine learning methods were Maximum Entropy (MaxEnt) [51], Genetic Algorithm for Rule Set Production (GARP) [52] and Artificial Neural Networks (ANN) [53]. These presence-only methods for modeling species' ecological niches can be grouped into three types of presence-only methods [54]. Firstly, into methods based solely on presence records (e.g. Euclidian and Gower distances), the prediction is made without reference to other samples from the study area. Secondly, into methods using “background” climatic data for the whole study area (e.g. ENFA, MaxEnt), which evaluate how the climatic conditions where species are known to occur relates to the climate across the rest of the study area (the ‘background’). Thirdly, into methods that generate (sample) “pseudo-absences” from the study area (e.g. GARP, ANN), assessing differences between occurrence sites and a set of sites chosen from the study area which are used instead of real absence data. In this case, the set of “pseudo-absences” were selected randomly [52].

For all models, we randomly partitioned presence and pseudo-absence data of each species in 75% for calibration (or training) and 25% for validation (or test); repeating this process 10 times (i.e. a cross-validation) and maintaining the observed prevalence of each species. We converted continuous predictions in presence and pseudo-absences finding the threshold with maximum sensitivity and specificity values in the receiver operating characteristic (ROC) curve and calculated the True Skill Statistics (TSS) to evaluate model performance [55]. The ROC Curve is created by plotting the fraction of true positives out of the positives vs. the fraction of false positives out of the negatives, at various threshold settings. The TSS range from −1 to +1, where values equal +1 is a perfect prediction and values equal to or less than zero is a prediction no better than random [55]. Although the area under the receiver operating characteristic curve (AUC) is the most common method to evaluate the accuracy of predictive distribution models, we decided to use TSS. There are several reasons why AUC should not be used for this purpose [56]. In particular, AUC weighs omission and commission errors equally and the total geographic extent of the study highly influences its scores [56].

We did an ensemble of forecasts to produce consensual predictions of species distributions [3], [10], [23], [28], [57]–[59]. We projected distributions to current climatic conditions and obtained 30 projections per species within each set of methods (3 modeling methods ×10 randomly partitioned data). We also projected distributions to future climate, obtaining 90 projections per species (3 modeling methods ×3 climate models ×10 randomly partitioned data). This allowed us to generate a frequency of projections in the ensemble. We then generated the frequency of projections weighted by the TSS statistics for each species and timeframe within each set of methods, i.e. best models have more weight in our consensus projections. We considered the presence of a species only in cells with 50% or more of frequency of projections, but a continuous value was held when this occurred. Then, we defined species richness as the sum of the ranges which overlapped (predicted by ENMs) for each cell. So, for example, if twenty different species were projected in a given cell the species richness for that cell was considered as twenty. Finally, we calculated species turnover between contemporary and future species distributions in each cell (G+L/S)/S+G, where “G” was the number of species gained, “L” the number of species lost and “S” is the contemporary species richness found in the cell.

### Evaluation of protected area effectiveness under climate change

The locations of the 187 protected areas (IUCN Categories I–IV) currently established in the Atlantic Forest were obtained from the United Nations Environmental Programme, World Conservation Monitoring Cemntre (UNEP-WCMC) [60]. These protected areas comprised 820 cells distributed in 11,461 Atlantic Forest cells. We overlaid protected area polygons onto our grid considering a grid cell as “protected” even if only a portion of it is protected, assuming that all species occurring in that cell could potentially benefit from the occurrence of a protected area in that cell.

Our aim was to evaluate whether current locations of protected areas are better than random allocations in protecting tiger moth diversity in the face of climate change. For this, we generated a null model that maintained size, form and orientation of protected areas but removed other intrinsic effects that likely will affect their suitability in the face of climate change (i.e. latitude, altitude). The null model allocated the protected areas randomly in the Atlantic Forest and obtained species richness in the present and the future based on the projections of species distribution models. Since there are many distinct possibilities to randomly allocate protected areas in the Atlantic Forest, this procedure was repeated 1,000 times and the average species richness obtained.

## Results

**Figure 2:**
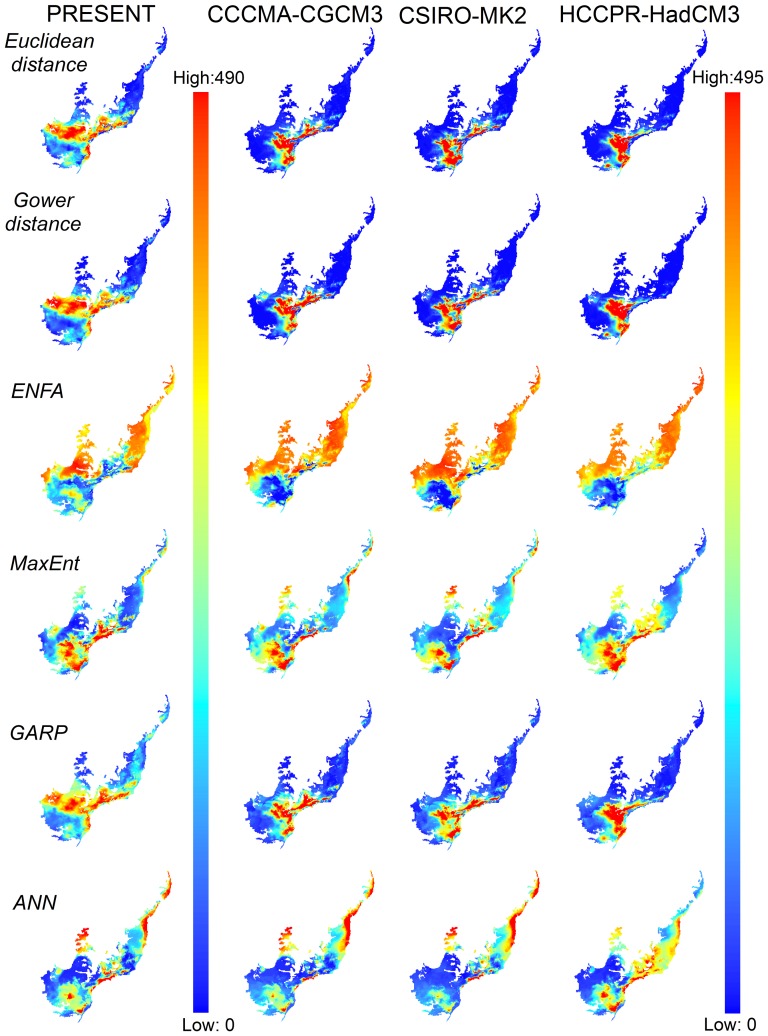
Species richness patterns for tiger moths in the Atlantic Forest Biodiversity Hotspot, Brazil. Tiger moth species richness patterns in the Atlantic Forest, Brazil (present and future, 2080, climate models CCCMA-CGCM3, CSIRO-MK2, and HCCPR-HadCM3) forecasted by ecological niche models generated by different distance modeling methods (Euclidian and Gower distances, Ecological Niche Factor Analysis, ENFA) and machine learning methods (Maximum Entropy, MaxEnt; Genetic algorithm for Rule set Production, GARP; Artificial Neural Networks, ANN).

**Figure 3:**
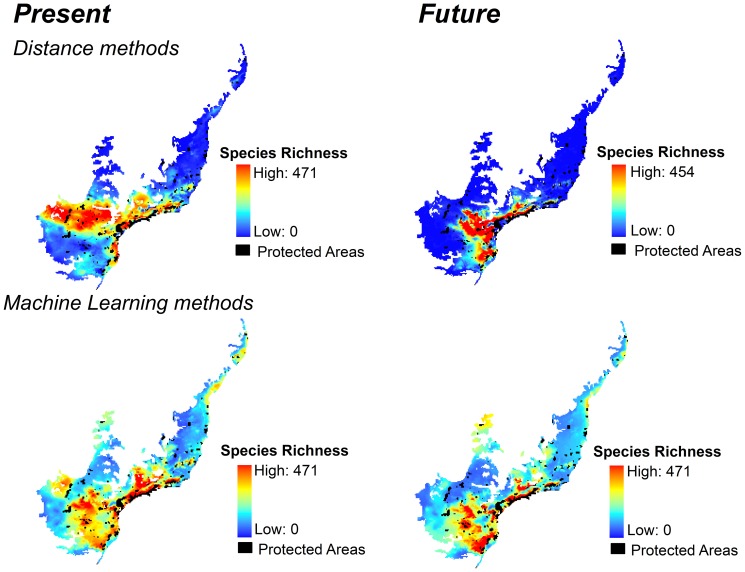
Consensus maps for tiger moth species richness in the Atlantic Forest Biodiversity Hotspot, Brazil. Maps of modeled tiger moth species richness based on consensus projections of 507 species predicted to occur in the Atlantic Forest Biodiversity Hotspot, Brazil, for current time (1950–2000) and 2080 (2051–2080) according to two different types of modeling methods and climate models. Models from distance and machine-learning methods were combined through an ensemble of forecasts to generate these consensus maps.

**Figure 4:**
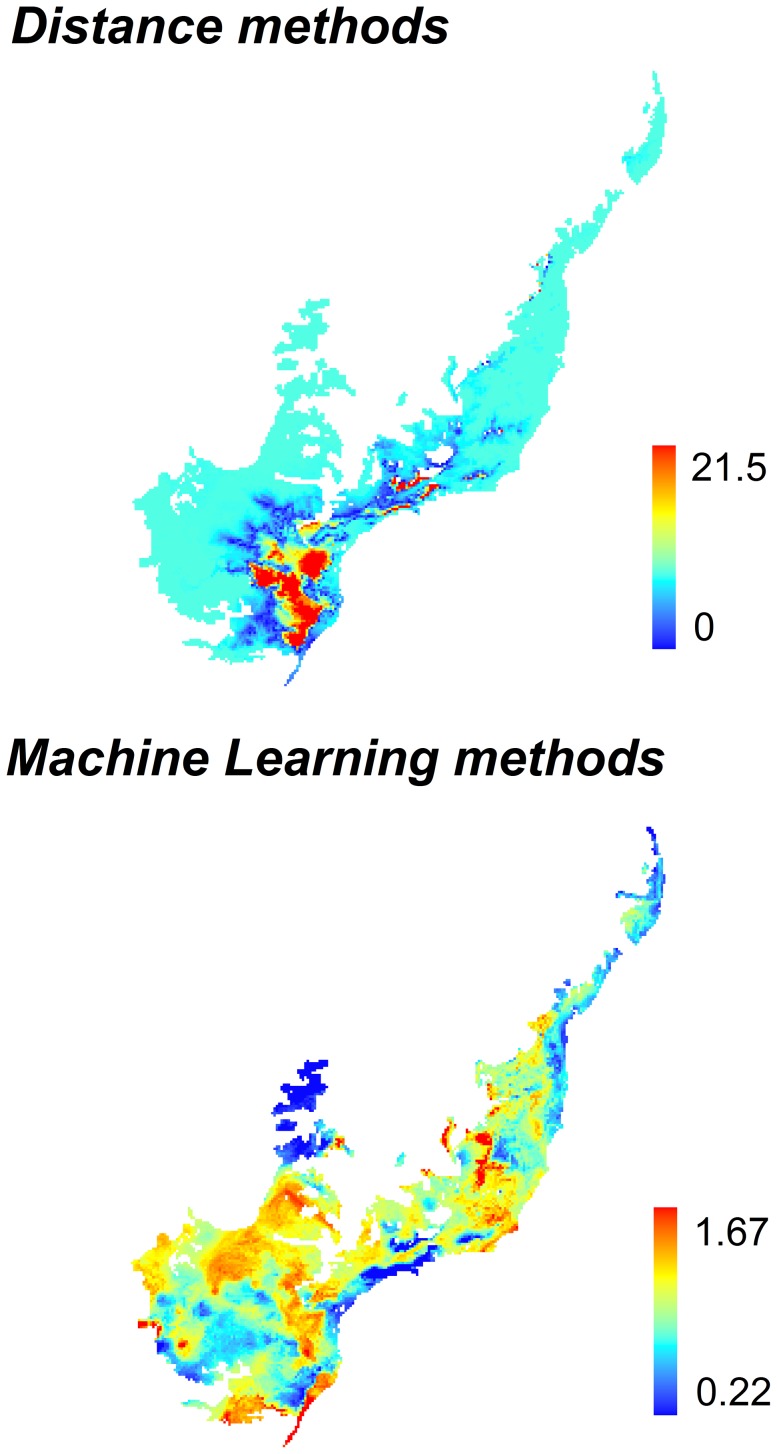
Species turnover pattern for tiger moths in the Atlantic Forest Biodiversity Hotspot, Brazil. Maps of modeled tiger moth species turnover based on consensus projections of 507 species predicted to occur in the Atlantic Forest Biodiversity Hotspot, Brazil, for current time (1950–2000) and 2080 (2051–2080) according to two different types of modeling methods and climate models. Models from distance and machine-learning methods were combined through an ensemble of forecasts to generate these consensus maps.

For most species, TSS values were relatively high (mean TSS ± SD = 0.55±0.05 for distance methods; and 0.64±0.14, for machine-learning methods) indicating good model fit (Table S1). Different modeling methods projected similar patterns of tiger moth species richness both for current and future climates, except for ENFA and ANN (Fig. 2). For all modeling methods, ensemble projections indicated areas of high species richness in the central and southern regions of the Atlantic Forest both for now and for 2080 (Fig. 3). However, these regions should become climatically unsuitable in 2080 for many species, decreasing species' distribution areas (Fig. 3, Table S2 and S3). We also found high species temporal turnover (up to 100% for all methods) across the projections between current and future climates (Fig. 4). This means that future scenarios showed dramatic changes not only in species richness but also in turnover of species composition (Table S4).

**Figure 5:**
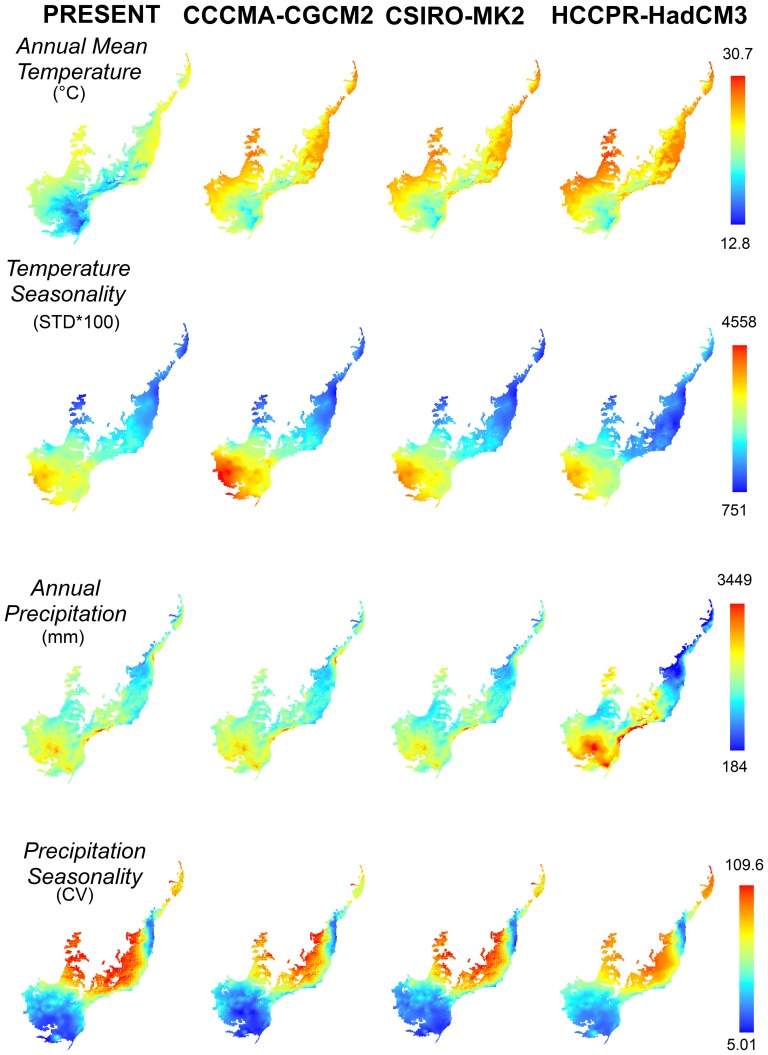
Expected changes for the four climatic variables used to model species' ecological niches in the Atlantic Forest Biodiversity Hotspot, Brazil. Maps show present conditions and values for future climate models CCCMA-CGCM3, CSIRO-MK2, and HCCPR-HadCM3, in 2080).

Central and northern sections of the Atlantic Forest should face higher temperatures, with lower seasonality, whereas the south should receive more rainfall, yet also have lower seasonality (Fig. 5). Most species had a significant range contraction (Table S3). Consensus of machine-learning methods projected up to 4.3% of species that had their ranges reduced by 100%, whereas consensus of distance methods projected 0.4% of species in the same situation (Table S3).

**Figure 6:**
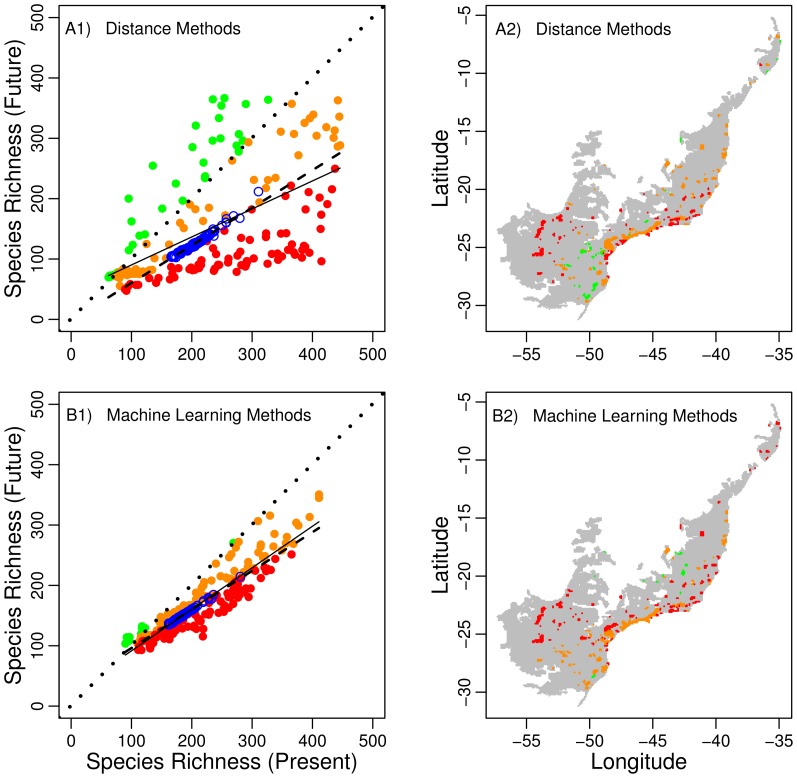
Relationship between present and future tiger moth species richness in the Atlantic Forest Biodiversity Hotspot, Brazil. Modeled present and future species richness of protected areas (filled circles) in the Atlantic Forest biome (A1 and B1). Open blue circles indicate expected species richness according to a null model of random location of protected areas in the biome. Dashed lines are the extrapolated regressions of the expected species richness according to the null model (A1: y∼−1.983+0.623*x; B1: y ∼32.178+0.640*x). Filled red circles indicate protected areas predicted to have severe species richness losses, defined as those in which future species richness will be lower than the predicted by a null model of random location of protected areas (below dashed regression line). Orange filled circles indicate protected areas predicted to have mild species richness losses, defined as those in which future species richness will be higher than the predicted by a null model of random location of protected areas. Green filled circles indicate protected areas predicted to gain species richness. Solid lines indicates the regression of modeled species richness in the future against modeled species richness in the present (A1: y∼43.817+0.464*x, R^2^ = 0.362, F_1,185_ = 105.2, P<0.001; B1: y∼22.736+0.688*x, R^2^ = 0.850, F_1,185_ = 1051.0, P<0.001). Maps of protected areas predicted to gain (green) or lose (orange, red) species in future changing climate (A2 and B2).

Future species richness should be lower than contemporary richness for most of the protected areas (Fig. 6). Differences between future and contemporary species richness were more varied for Distance than Machine Learning methods (Fig. 6A1 and 6B1). Distance models show a more optimistic result, with some protected areas holding more species (green dots in Fig. 6A1 and protected areas in the Fig. 6A2) in the future than in current time. These protected areas predicted to gain species richness are mostly located in the mountainous southeastern portion of the biome.

Machine-learning methods predicted that almost all protected areas will lose species in the future (Figs. 6B1 and 6B2). The main expectation of the null model of random location of protected areas is the loss of species in the future, both for Distance (Fig. 6A1) and Machine Learning (Fig. 6B1) methods. Among the protected areas predicted to lose species (orange and red dots and protected areas in Fig. 6), around half of them should experience losses worse than those predicted by the null model (red dots and protected areas in Fig. 6). These protected areas are concentrated in the southwestern and northern sections of the biome.

In addition to the general trend of species loss, the baseline comparison provided by the null model of random allocation of protected areas (blue dots) indicated great variation between how many species protected areas would lose (and also gain in case of distance methods). For machine-learning methods, species richness for current and future climates for the real locations of protected areas was generally lower (red dots and protected areas in Fig. 6) than those obtained from the random locations of protected areas (blue dots in Fig. 6). Nearly half of currently established protected areas should become climatically unsuitable at rates higher than expected by a random distribution of protected areas (red dots and protected areas in Fig. 6).

Protected areas predicted to gain species (particularly for distance methods) are mostly located in the cooler southern region of the Atlantic Forest (Fig. 6). Protected areas along the coast or in adjacent mountainous areas should lose species, although those in the north would do so at rates higher than those expected by our null model (protected areas indicated in red). The western region of the biome, which includes semideciduous forests, will experience a severe reduction in species richness due to climate change (Fig. 6).

## Discussion

We forecasted species' range shifts and range contractions for most tiger moth species inhabiting the Atlantic Forest to evaluate the effectiveness of existing protected areas [61], [62]. Our findings indicate that most protected areas should become climatically unsuitable for sustaining their current number of species under climate change. As Atlantic Forest protected areas will become less effective in safeguarding moths, it is important to anticipate how climatic changes will lead to a decreasing species representation across the entire network of protected areas.

Our results agree with Pearson and colleagues [63] which detected similar accuracy among species distribution models, although the spatial pattern in the predictions was different. Distance methods are simple methods that do not consider complex relationships between species occurrence and predictors, use presence-only data and tend to underestimate the distribution in novel conditions like those expected to occur under climate change [24], [63]. Alternatively, machine-learning methods are very complex, assume different relationships and can underestimate or overestimate distributions in novel conditions [24], [63]. These features help explain the differences in our results obtained by distance methods when compared with machine-learning methods, especially regarding the pattern of species richness and turnover rates.

The choice of a perfect set of modeling methods, however, is not an easy task. When predicting climate change effects on species distributions, commission errors lead to the overestimation of range expansions whereas omission errors produce overestimates of range contractions. If one is employing such models to predict regions of climatic stability to guide conservation actions, both omission and commission errors are of particular interest, as these errors are likely to produce huge bias in the results of gap-analysis as well. Distance models tend to inflate commission errors and individual species distribution, which are usually larger than predicted by other models. Machine-learning models usually result in smaller distributions when compared to other models. Thus, if conservation practitioners want to reduce commission errors, machine-learning models are perhaps the best option. Although, in this case, one will probably face the inconvenience of a lack of clarity and in turn, a difficulty of explaining results to stakeholders and decision makers involved in the conservation process [27].

Our study predicts that up to 4.3% of tiger moth species should face 100% of range contraction in the Atlantic Forest's future. Among these species that would disappear from the region, three of them are endemic (*Clemensia marmorata*, *Cosmosoma cingla*, and Arctiini NI92 morphospecies). The majority (n = 18) of species predicted to have a 100% of range contraction belong to the Arctiini tribe (in general robust, medium-sized moths and polyphagous larvae) and the Lithosiini tribe (in general slender, small-sized moths, and lichen-feeding larvae). Unfortunately, there islittle, scattered information on the natural history of and the appropriate climatic conditions for these species available (as for the vast majority of Neotropical species). It is therefore hard to make recomendations for conservation strategies for these species.

In general, the consequences of these extinctions can spread through networks of interaction causing extinction cascades and consequent disrupted ecological functions in ecosystems [19], [64]. In addition to causing local and regional extinctions, climate change can alter the size and location of the range and patterns of abundance and phenology of species [38]. Many lepidopterans, including Arctiinae species [65], are agricultural pests and changes in range, abundance and phenology of these species may increase the rate of invasion and increase the damage intensity they can cause [66], [67]. Furthermore, climate change can modify patterns of synchronization between larvae and host plants, among adults and flowers, and among the two life stages and their natural enemies [68]. Some tiger moth species are important pollinators of Atlantic Forest plants, including orchids [69], and the lack of synchronization between flowering and pollinating may cause more extinctions.

We also found an increase in species richness in the future in a few southern areas of the biome, due to the expansion of the range toward higher latitudes. This pattern has been observed in studies on Lepidoptera [9], [70] and other groups [8]. It is known, however, that species with wide latitudinal ranges could be preadapted to cope with climate change because they already find considerable temperature variation across their habitats. However, low dispersal capability and difficulties to cross open habiats may prevent or at least slow down range shifts of Arctiinae [38]. For instance, several studies have found that Arctiinae species disperse over short distances in natural conditions [71], [72]. The average distance traveled by *Dysauxes ancilla* L. moths in two consecutive days (at mark-released-recapture study), for example, was only 43 m [72]. The same author observed that moths rarely leave their breeding area and did not cross very open areas or dense forest. In fact, two other studies in Brazilian Biodiversity Hotspots, the Atlantic Forest [73] and the Cerrado [74] found that Arctiinae fauna of open vegetation (grassland) differed from the denser vegetation (forest) and a very small number of species co-occurred in these vegetation types. More importantly, these differences were evident even when the sample sites were close to each other (about 100 m). Accordingly, the low dispersal capability of the species of Arctiinae compounded with the low ability of the species to cross open areas in fragmentated landscapes of the Atlantic Forest should further complicate the future of these species. This is particularly concerning in the Atlantic Forest, given that human activity has degraded more than 85% of the biome [47].

One strategy to mitigate the loss of species in the Atlantic Forest protected areas in the future would be the creation of protected areas in southern regions and at higher altitudes of the Atlantic Forest. Klorvuttimontara and colleagues [75], for example, despite having recorded a reduction of approximately 30% of butterfly richness in the protected areas of Thailand in the future (A2 scenario), observed that the effectiveness of protected areas remained almost the same in the future. The explanation for this was that most protected areas in Thailand are located at high altitudes, allowing the butterflies to migrate from lowland areas to higher altitudes as the climate becomes unsuitable in the future. Of course, critical questions should be addressed to actually implement a decision like this, such as infostering the implementation of protected areas in high-altitude regions. Some questions would be, for example: what are the indications that these regions would protect other taxa than moths? Weel, it seems defensible to predict that they likely will be, because many species and communities are likely to migrate south and to higher elevations in response to climate change [7], [8], [10], [17]. Should protected areas be placed to span altitudinal ranges within the protected area itself, so that species can start low and move steadily higher as the climate changes? These questions are still difficult to answer, but we hope our study is a step towards scientifically driven decisions in future protected area establishment and management.

The establishment of protected areas is still one of our best conservation actions to protect biodiversity. The Atlantic Forest has been indicated as aconservation priority by several world studies using different criteria [76], [77]. Our approach is one of the first to incorporate climate change threats on the long-term assessment of protected areas (see [5], [17], [21], [77], [78]). In fact, climatic changes are increasingly driving species out of protected areas due to species range shifts [5], [79], [80].

It is important to highlight some of the caveats of our study. Firstly, our database has a large number of records, which were gathered from field studies and museum records. Although the data is dense and trustworthy, there could be some bias towards easily assessed sampling regions or toward large, colorful species, as in all kinds of samplings. Further, some records were from as early as 1920 and climate has likely shifted already since then. Secondly, our models assume tiger moths are in equilibrium with the current climate and have unlimited dispersal to tackle suitable climates as they move in the geographic space. These are simple assumptions allowing us to model all species distribution at a time. Thirdly, we predicted future species distribution assuming that the vegetation types in the Atlantic Forest will remain in the same regions of the current distribution. This last assumption can affect our species distribution model predictions. Species' range shifts outside of the current limits of the biome or their preferential habitat cannot be measured by our methods. Fourthly, the effectiveness of protected areas tends to be overestimated given that species were considered to be protected if any part of their range overlapped with protected areas polygons [81]. However, simple presence within a protected area is insufficient to ensure the long-term persistence of many species. Most existing protected areas in the Atlantic Forest were created without following any ecological criterion and species representation in this network is highly variable. Clearly, there is a need to update the current protected areas to improve species representation and climate change is an important factor to be considered.

As a final message, it is important to remember that species will generally alter their distributions independently. How do we prioritize which species to try to track through these changes? In this paper we recognize this conundrum and selected Tiger moths as our model group because of available data and diversity of species. It is now cear that we simply can't model or predict the response of all species on Earth to climate change. However, there are some generalities, such as the general tendency of species to mode southward or uphill in the southern hemisphere that could be helpful, if combined with models like the one we presented here, to guiding decisions about how to better allocate finite resources for biodiversity conservation.

## Supporting Information

Table S1 Tiger moth tribes, species, True Skill Statistics (TSS) for each ecological niche modeling method and the ensembles among distance methods (Euclidian and Gower distance and ENFA) and machine-learning methods (MaxEnt, GARP, and Artificial Neural Networks).
(XLS)

Table S2 Tiger moth tribes, species, and number of grid cells predicted to be occupied during baseline climate (1950–2000) and future climate (2051–2080) under different climate models and ecological niche modeling methods.
(XLS)

Table S3 Tiger moth tribes, species, and percent of range contraction predicted to during baseline climate (1950–2000) and future climate (2051–2080) under different climate models and ecological niche modeling methods.
(XLS)

Table S4 Turnover measures for each of 11,464 grid cells overlaying the Atlantic Forest, Brazil, in this study. Turnover is shown for each modeling method (Euclidian and Gower distance, ENFA, MaxEnt, GARP, and Artificial Neural Networks), and climate model (CCCMA-CGCM, CSIRO-MK2, and HCCPR-HadCM3). Turnover found across the ensemble of distance and machine learning methods are also shown.
(XLS)
